# Effect of the combination of biological, chemical control and agronomic technique in integrated management pea root rot and its productivity

**DOI:** 10.1038/s41598-022-15580-1

**Published:** 2022-07-05

**Authors:** Nargis Nazir, Zaffar Afroz Badri, Nazir Ahmad Bhat, Farooq Ahmad Bhat, Phalisteen Sultan, Tashooq Ahmad Bhat, Mohd Ashraf Rather, Aafreen Sakina

**Affiliations:** 1 Division of Plant Pathology, Faculty of Agriculture, SKUAST- K, Wadura, Sopore, Jammu and Kashmir 193201 India; 2 Krishi Vigyan Kendra Malangpora, Pulwama, Jammu and Kashmir 192301 India; 3 Mountain Research Centre for Field Crops, Khudwani, Kulgam, Jammu and Kashmir 192101 India; 4 CSIR-IIIM, Srinagar, Jammu and Kashmir 190005 India; 5 Division of Food Science and Technology, SKUAST-K, Shalimar, 190025 India; 6 Division of Fish Genetics and Biotechnology, Faculty of Fisheries, SKUAST-K, Rangil, Ganderbal, 191201 India

**Keywords:** Fungi, Fungal pathogenesis

## Abstract

Root rot of pea caused by *Fusarium* spp. is one of the important diseases of pea (*Pisum sativum* L.). The causal fungus of the disease isolated from naturally infected pea plants was identified as *Fusarium solani* f. sp. *pisi* (Jones). Evaluation of four bio agents and nine fungicides was done in vitro against *Fusarium solani*. *Trichoderma harzianum* was the most effective bio agent in inhibiting the mycelial growth of *F. solani* by (82.62%). Carbendazim 50 WP was the most effective fungicide in inhibiting the mycelial growth of *F. solani* by (91.06%). Carbendazim at the rate of 0.1% and *T. harzianum* at concentration of 10^9^ cfu when used as seed treatment under field conditions were evaluated along with three planting techniques v.i.z, raised beds, ridges and flat beds. It was found that Carbendazim at the rate of 0.1% when given as seed treatment in raised beds exhibited the lowest disease incidence (10.97%), intensity (2.89%) and the maximum pod yield (89.63 q ha^−1^) as compared to control.

## Introduction

Garden pea (*Pisum sativum* L.) is an important cool season annual legume crop whose origin can be traced back to the Middle East. The crop is rich source of vitamins and minerals like Ca and Mg. It also has a high quantity of fiber that improves bowel health. Pea also contains Vitamin B complex (Niacin) that helps in the reduction of triglycerides, thereby resulting in less cholesterol. Further different compounds present in pea like coumestrol, pisum saponins I & II and phenolic acids help in the prevention of stomach cancer. India is the largest producer of pea in the world with a production & productivity of 48.11 lakh tones and 9.0 t ha^−1^ respectively^1^. In India, Uttar Pradesh stands at the no. 1 in production of pea. Despite its high nutritional value and remarkable production, the yield of the crop gets drastically constrained due to certain diseases like root rot, powdery mildew, fusarium wilt etc.

Root rot of pea caused by *Fusarium solani* is often considered a major constraint in pea production worldwide^2^. It causes severe damage at all stages of crop growth and upto 97 per cent yield losses were reported by El-Saadony et al.^3^. In India, root rot of pea was first reported by Sukapura et al.^4^ from Pune. The disease has also been reported from Kashmir valley with an incidence ranging from 14.8 to 64.7 per cent^5^. Reddish brown streaks of the roots near the cotyledon attachment point is among the first signs of fusarium root rot. As the streaks coalesce, they form a black lesion that encircles the roots and epicotyls. The roots of infected plants become dark and weak as the root rot progresses, and they commonly disintegrate when they are removed from the soil. Infected populations in a crop may appear healthy for a short time before unexpectedly collapsing, especially if hot, dry weather comes during pod filling, when pea plants are especially vulnerable to moisture stress^6^. Above ground symptoms of plant infected by *Fusarium solani* f. sp. *pisi* can be characterized by yellowing of leaves, starting at the base of the plant which later on progresses to the top of the plant^7^. Wilting or death of infected plants is not seen, but the growth of the infected plant can be drastically stunted.

Root rot of pea is a serious threat to profitable cultivation of pea, efforts have been made worldwide to manage the disease through chemicals^8^, bio agents^9^ and cultural interventions^10^. Available literature, however, does not reveal any work conducted on the effect of the above-mentioned management practices in combination. Therefore, looking into the importance of the crop and the disease, the present investigation was taken up to develop a suitable and sustainable strategy to control this disease and reduce the yield losses through an integrated approach.

## Materials and methods

### Raw material

The raw material was procured from the registered centers of Faculty of Agriculture Wadura, SKUAST-K, India & all the methods used in this work are in compliance with institutional guidelines. All chemicals used for analysis were obtained from Sigma Aldrich.

### Symptomatology

Symptomatology was carried out on pea plants showing typical symptoms of root rot (Thoroughly identified by Division of Plant Pathology SKUAST-K). The diseased plants showing above ground symptoms in the field during the course of survey were bought to the laboratory. The roots were washed using tap water before making observations for root rot symptoms. Symptomatic plants were maintained under natural conditions in field to record periodic symptom development vis-à-vis root colour, tissue disintegration and its effect on aerial parts.

### Isolation of pathogen

The tissue bit transfer approach was used to isolate the causative agent^11^. With a sharp sterilised blade, the symptomatic diseased roots were sliced into little bits (2–3 mm) such that each sick bit contained a portion of healthy tissue. These parts were surface sterilised for 30 s with a 0.1% mercuric chloride solution, then rinsed three times with distilled sterilised water to eliminate any remaining mercuric chloride solution. The bits were blotter dried before being aseptically transferred to Potato Dextrose Agar (PDA) media in sterile Petri-plates and incubated at 25 ± 1 °C and examined periodically the color of mycelium or colony.

### Purification of pathogen

To achieve axenic culture of the pathogen, the single spore or hyphal tip approach described by Xing et al.^12^ was used. Fungal growth observed on diseased tissue bits was aseptically transferred to Petri plates containing PDA and incubated at 25 ± 1 °C for 7 days. The sub cultured plates were then observed for sporulation. Dilute spore suspension in sterile distilled water, prepared out of a sporulating colony was poured on Petri plates containing water agar and incubated for 1 day at 25 ± 1 °C. These water agar plates were then observed in inverted position under microscope and the isolated germinated spores were transferred to fresh plates containing PDA and incubated at 25 ± 1 °C. Pure obtained cultures were stored at 5 °C for further use. Identification of the isolated fungi was carried out according to their cultural, morphological and microscopic characteristics as described by Barnett and Hunter^13^.

### Identification of pathogen

The pathogenic isolate on pea plants was identified on the basis of morphological characters of somatic and reproductive structures and compared with the monograph on *Fusarium* spp by Aksoy et al.^14^.

### Pathogenicity test

El-Dawy et al.^15^ technique was used to perform the pathogenicity test. One-third portion of root system of 10–13 days old seedlings was clipped off from distal end and dipped for 5 min in conidial suspension prepared from the spores of purified fungal culture isolated from root rot affected pea plants. The inoculated plants were transplanted back in sterile soil containing pots. Clipped plants were dipped in sterile distilled water for the same period of time in case of control. Observations on development of typical symptoms on the inoculated plants were made seven days after inoculation.

### In vitro evaluation of bio-agents

Bio-agents i.e. *Trichoderma harzianum*, *T. viride*, *Bacillus subtillus* and *Pseudomonas fluorescens* were procured from bio-fertilizer lab of FoA Wadura SKUAST-K. The bio-agents were then cultured on PDA and allowed to grow at 25 ± 2 °C for ten days and preserved in refrigerator for in vitro and in vivo studies. The bio-control agents were then evaluated against *Fusarium* spp. through dual culture method^16^. Culture discs (5 mm) of each fungal antagonist and the pathogen were taken from the margin of the actively growing cultures and transferred to PDA medium in 90 mm petri plates on opposite side, approximately at 10 mm from the wall of the plate while bacterial bio agents were streaked on the opposite side of the pathogen. A check having the pathogen only was maintained under similar conditions for comparison. The petri plates were subsequently incubated at 25 ± 10 °C till mycelial inhibition was observed. Colony diameter of the fungus in the dual culture as well as of the test fungus was recorded. Per cent growth inhibition of the pathogen over control was calculated according to the formula given by Kipkoech et al.^17^ as:

1 $${\text{I}} = \frac{{{\text{C}} - {\text{T}}}}{{\text{C}}} \times 100$$

where I = % Inhibition; C = Colony diameter in control T = Colony diameter in treatment.

### In vitro evaluation of fungicides

Nine fungicides viz*.,* Carbendazim 50WP, Captan 50WP, Hexaconazole 5EC, Difenconazole 25EC, Tebuconazole 75WG, Kresoxim methyl 44.3SC, Pyraclostrobin 60WG, Mancozeb 75WP and Metalaxyl 72WP were evaluated against the pathogen by poisoned food technique^18^. Potato Dextrose Agar Medium (PDA) was prepared and sterilized at 121 °C for 20 min. Simultaneously, several fungicide concentrations were prepared in sterilized distilled water. Before pouring in Petri plates, appropriate volumes of fungicide solution were added separately to equal quantities of PDA medium in an aseptic manner. After that, the plates were injected with a 7-day-old test pathogen. In addition, a control was kept in which only ordinary sterilized water was added to the PDA medium. Each treatment was repeated three times, and inoculation plates were incubated in a Bio-Oxygen Demand (BOD) incubator at 25 ± 1 °C. Using the formula above, the percentage in inhibition of mycelial growth at different test concentrations in comparison with control treatment.

### Management of the disease in field

In vivo experiment was conducted during Rabi season (October- November) at Faculty of Agriculture, SKUAST-K, Wadura. The experiment was laid in Randomized Complete Block Design with two factors viz., seed treatment and planting methods. The fungicide (Carbendazim) and bio agent (*Trichoderma harzianum*) that proved most effective in vitro were used as seed treatments under field conditions against root rot of pea. Field was equally divided into raised beds, ridges and flat beds with a plot size of 2.1 m × 2.1 m with each plot having 50 plants was maintained and replicated thrice. The pea seeds of variety Arkel were treated with carbendazim or *T.harzianum* before sowing. In case of seed treatment with carbendazim, the seeds were slightly moistened with sterile water and carbendazim at the rate of 1 g kg^−1^ seeds was added in a round bottomed plastic vessel. The vessel was shacked vigorously till a thin film of fungicide was layered on all seeds. The seeds thus treated were dried in shade before sowing. In case of seed treatment with *T. harzianum*, a spore suspension of the bioagent was prepared and diluted to 10^9^ cfu (50 ml kg^−1^). The seeds were immersed in the suspension for 30 min and later dried in shade. In case of control plots, seeds were treated with sterile water only.

## Results and discussions

### Symptoms

Detailed symptomatology vis-à-vis underground and above ground sufferings of affected pea plants were recorded and the observations are described as under:

#### Roots

**Figure 1:**
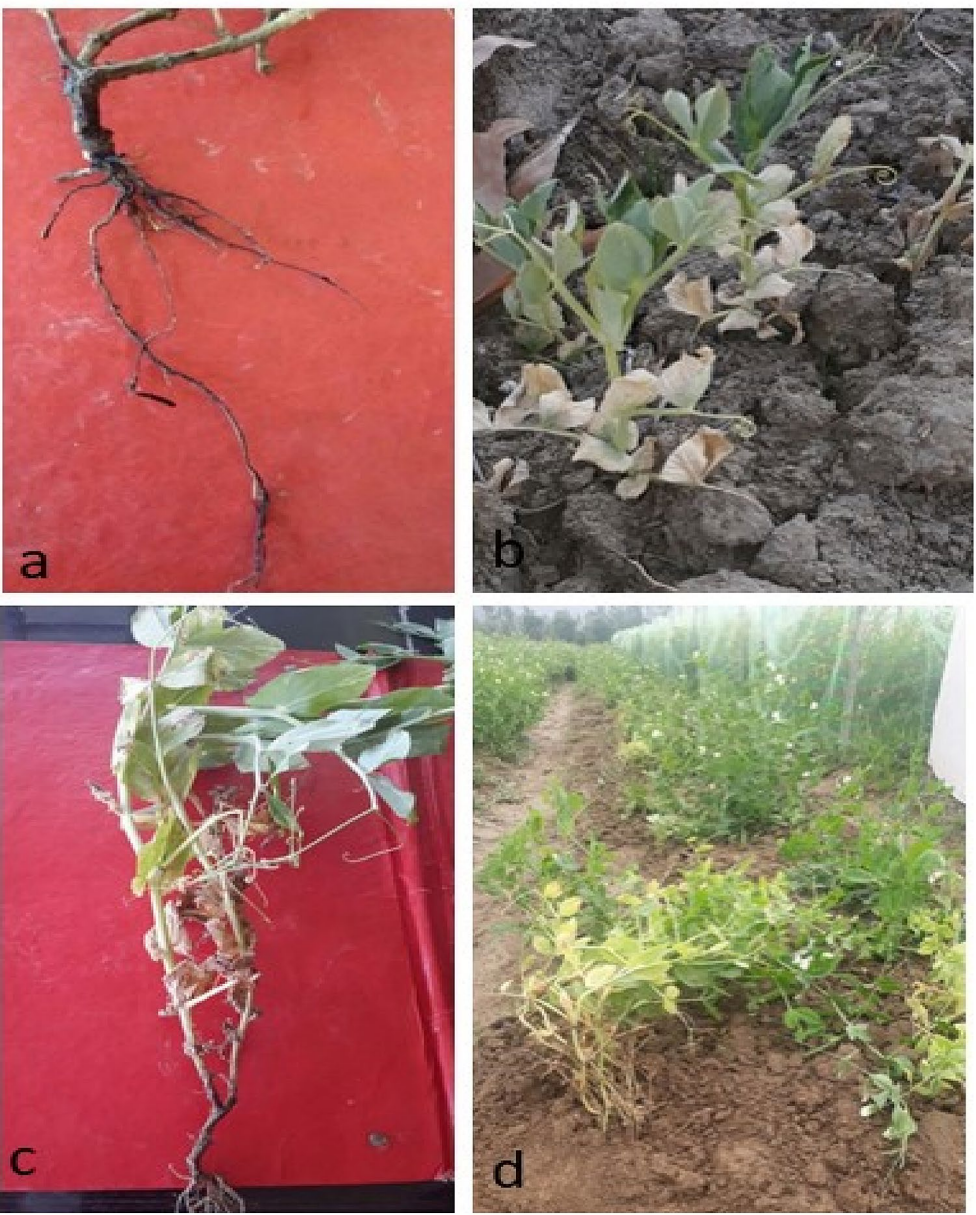
Symptoms of root rot of pea. (**a**) Blackish to brownish streaks on roots, (**b**) yellowing of lower leaves, (**c**) wilting and stunting of the lower leaves, (**d**) epinasty shown by diseased pea plants.

Roots affected with root rot showed reddish brown lesions as initial symptoms near the soil line and below it. As the disease progressed, these lesions became dark brown to black, colleased together and spread continuously throughout the roots (Fig. 1a). Lateral roots were reduced with distorted root hairs. Diseased roots were sloughed and macerated. When the roots showing initial symptoms were cut longitudinally, no vascular discoloration was observed. However, in advanced phase of disease, when there was extensive tissue disintegration, the discoloration had advanced to the interior of the roots as well. Similar inferences were drawn by Porter et al*.*^19^.

#### Above ground symptoms

Root rot affected plants showed yellowing of the lower leaves which later progressed towards the top (Fig. 1b). As the disease became more aggressive, the lower leaves appeared wilted (Fig. 1c). Root rot affected plants also showed epinasty (Fig. 1d).

### Isolation of pathogen

**Figure 2:**
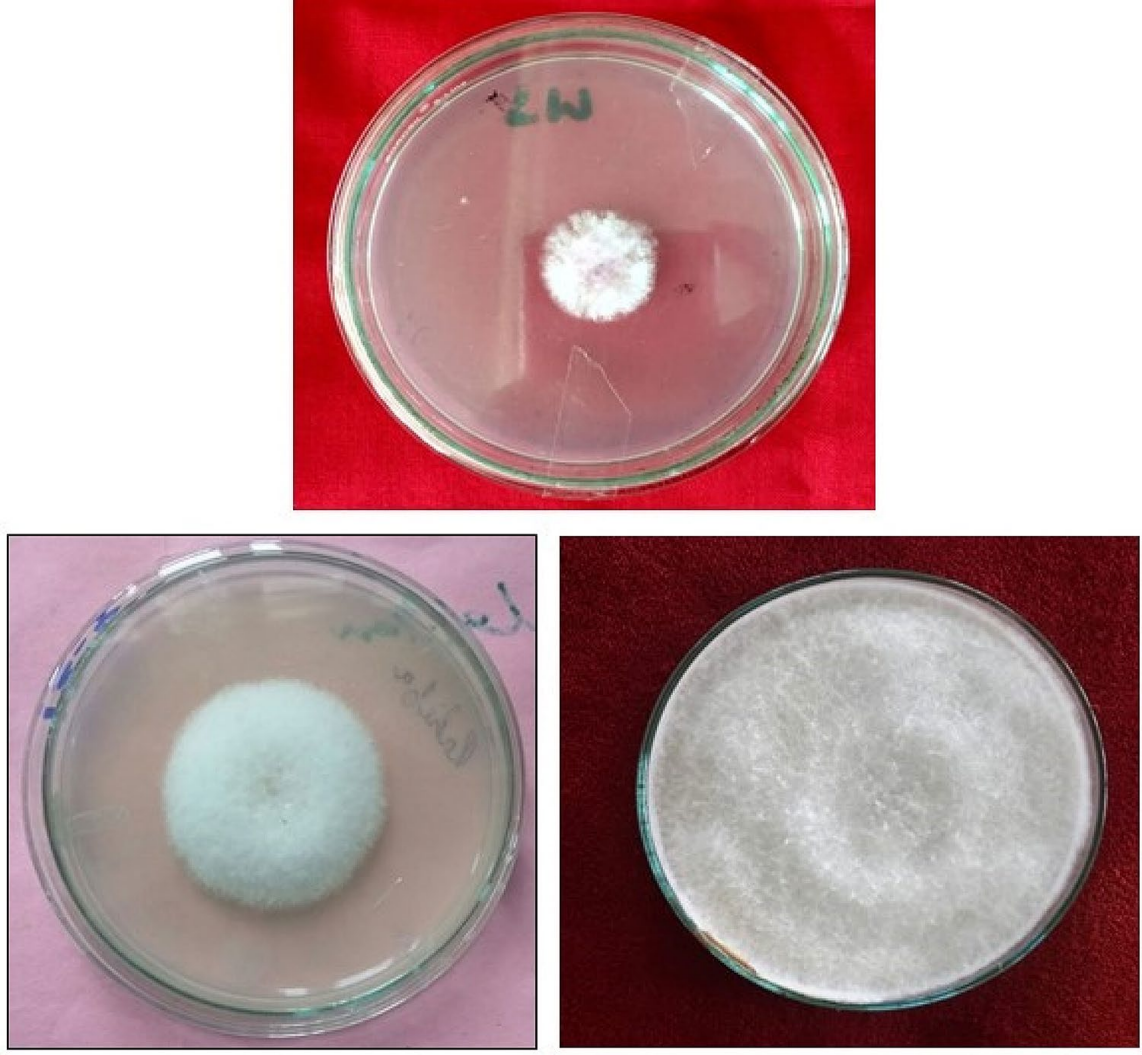
Isolation, purification and maintenance of the pathogen.

In the present investigation, *F*. *solani* was isolated from the black-brown, decaying infected roots of pea. The fungus produced scanty aerial mycelium having puff pink cottony growth with 30–36 mm diameter in 3 days (Fig. 2).

### Identification of pathogen

**Figure 3:**
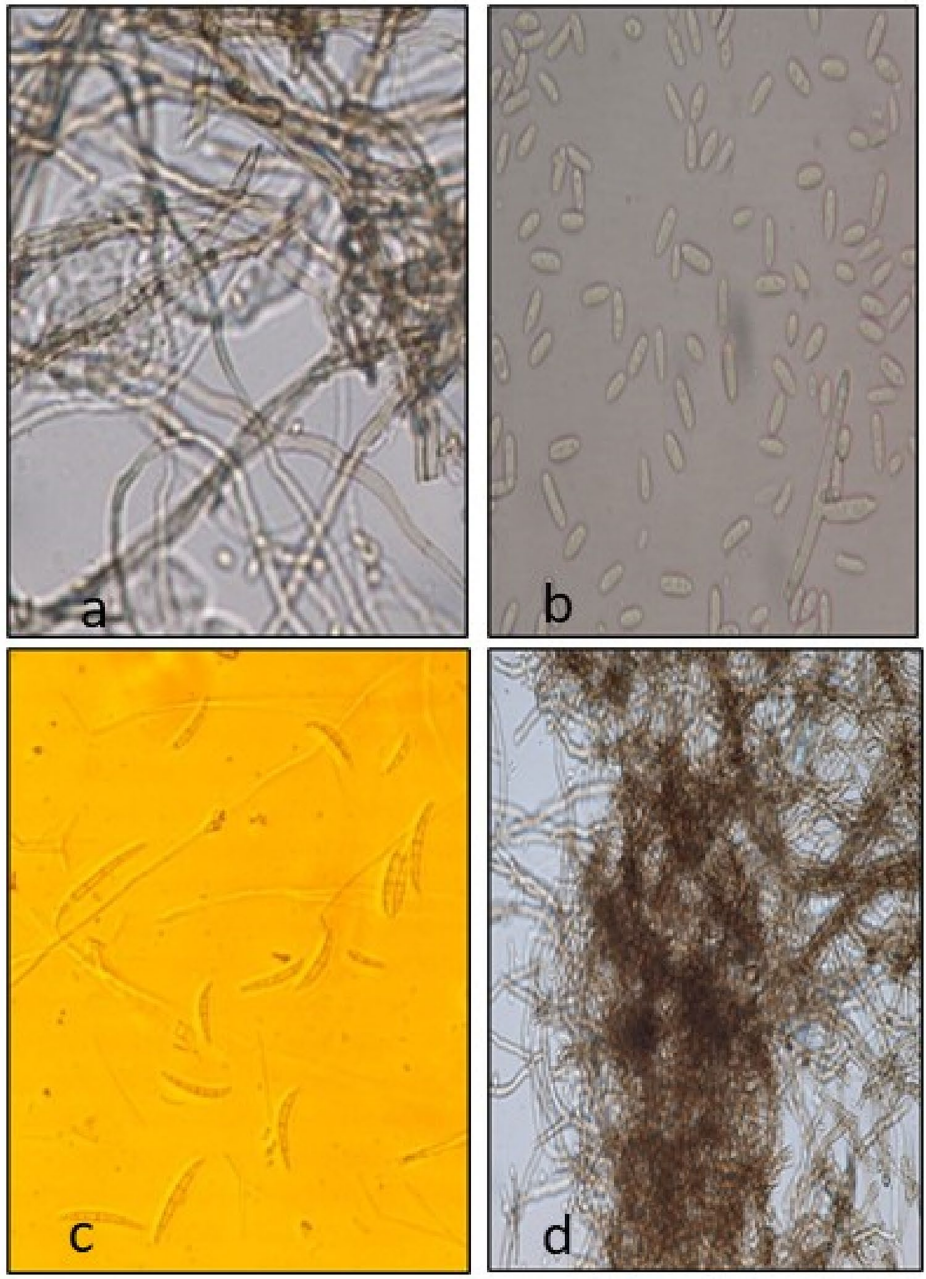
Morphological characteristics of the pathogen. (**a**) Mycelium, (**b**) micro conidia, (**c**) macro conidia, (**d**) chlamydospores.

The morphological characteristics of the fungus on PDA medium is given in Table 1, Fig. 3. The study revealed that the mycelium was septate and branched and the colonies appeared white to creamy. Macro conidia were 4–5 septate, fusiform and curved measuring 32–41 × 5–7 µm. Micro conidia were abundantly present, oval to ellipsoid in shape measuring 12–17.5 × 2.5–4.5 µm. On the basis of morphological and colony characters and the monograph by Aksoy et al.^14^, the fungus was identified as *F*u*sarium solani* (Jones). Similar morphological characters were reported by Kumari et al*.*^20^.

**Table 1:** Morpho-cultural characteristics of isolated pathogen.

Thallus part	Shape and physical appearance	Colour	Size (µm)*	Septation
Colony	Fast growing and circular	Initially dull whitish to creamish, later puff pink in growth	80 mm after 7 days of incubation	–
Mycellium	Hyaline, septate and branched	Hyaline	Hyphal width 1.5–2.0	Present
Macroconidia	Fusiform to curved	Brown	32–41 (33.5) × 5–7 (4.0)**	4–5 septate
Microconidia	Oval to ellipsoid	Brown	12–17 (9.5) × 2.5–4.5 (3.2)	Aseptate
Chylamadospores	Globuse, single celled	Brown	9.0 × 6.60	–

*Values are means of 15 replications.

**values in parenthesis are mean values.

### In vitro evaluation of bio agents

**Figure 4:**
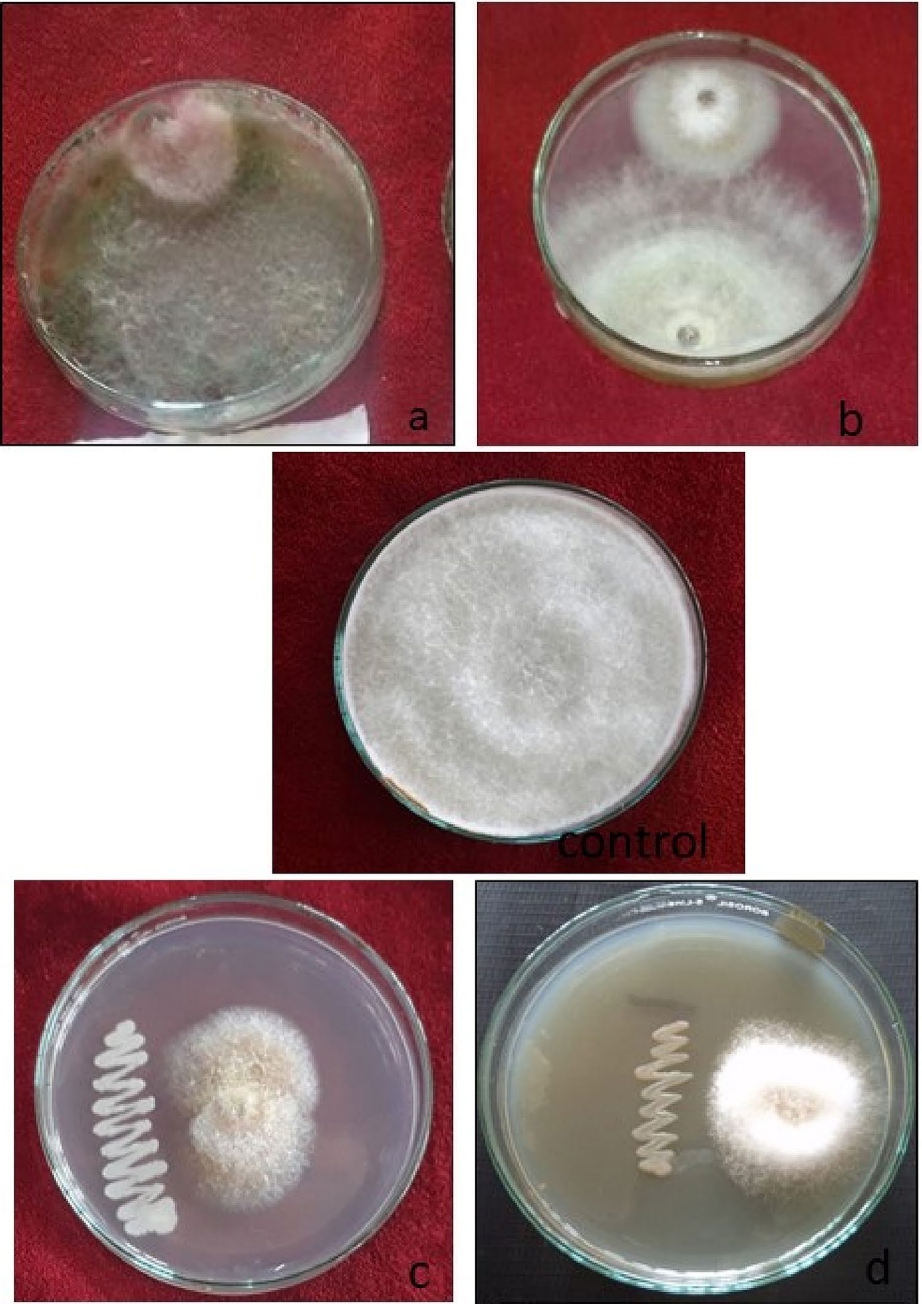
In vitro efficacy of bio agents against pathogen mycelial growth. (**a**) *T. harzianum*, (**b**) *T. viride*, (**c**) *B. subtilis*, (**d**) *P. fluorescens.*

It is evident from the data obtained that all the bio agents proved antagonistic against *Fusarium solani* (Table 2, Fig. 4). Minimum colony diameter of pathogen (14.25 mm) was recorded in dual culture with *Trichoderma harzianum* as compared to control, where a colony diameter of 82.00 mm was recorded after 7 days. The pathogen showed a colony diameter of 17.75, 23.25 and 28.00 mm in dual culture with *T*. *viride*, *B*. *subtilis* and *P*. *fluorescens*, respectively. Thus, *T. harzianum* was found to be superior over all treatments with 82.62 per cent growth inhibition followed by *T. viride* (78.35%), *B. subtilis* (71.64%) and *P. fluorescens* (65.85%). Similar results were shown by Ahmed et al.^21^ and Hamid et al.^22^. The mechanism of bio control adopted by bio agents may be lysis, competition, hyper parasitism and possessing some important secondary metabolites like viridian, harzianol etc^23^. Bio control agents are able to survive and consume nutrient sources more rapidly by outcompeting the pathogens, thereby declining their population^24^. Further spp. like Trichoderma has been reported to produce iron-binding siderophores that control the Fusarium wilt^25^.

**Table 2:** In vitro efficacy of various bio-agents on mycelial growth inhibition of *Fusarium solani.*

S. no.	Bio-agent	Colony diameter (mm)	Mycelial growth of pathogen over check (%)
1	*T*. *harzianum*	14.25	82.62
2	*T*. *viride*	17.75	78.35
3	*B*. *subtilis*	23.25	71.64
4	*P*. *fluorescens*	28.00	65.85
5	Control	82.00	–

C.D (*p* ≤ 0.05) = 1.48.

### In vitro evaluation of fungicides

**Figure 5:**
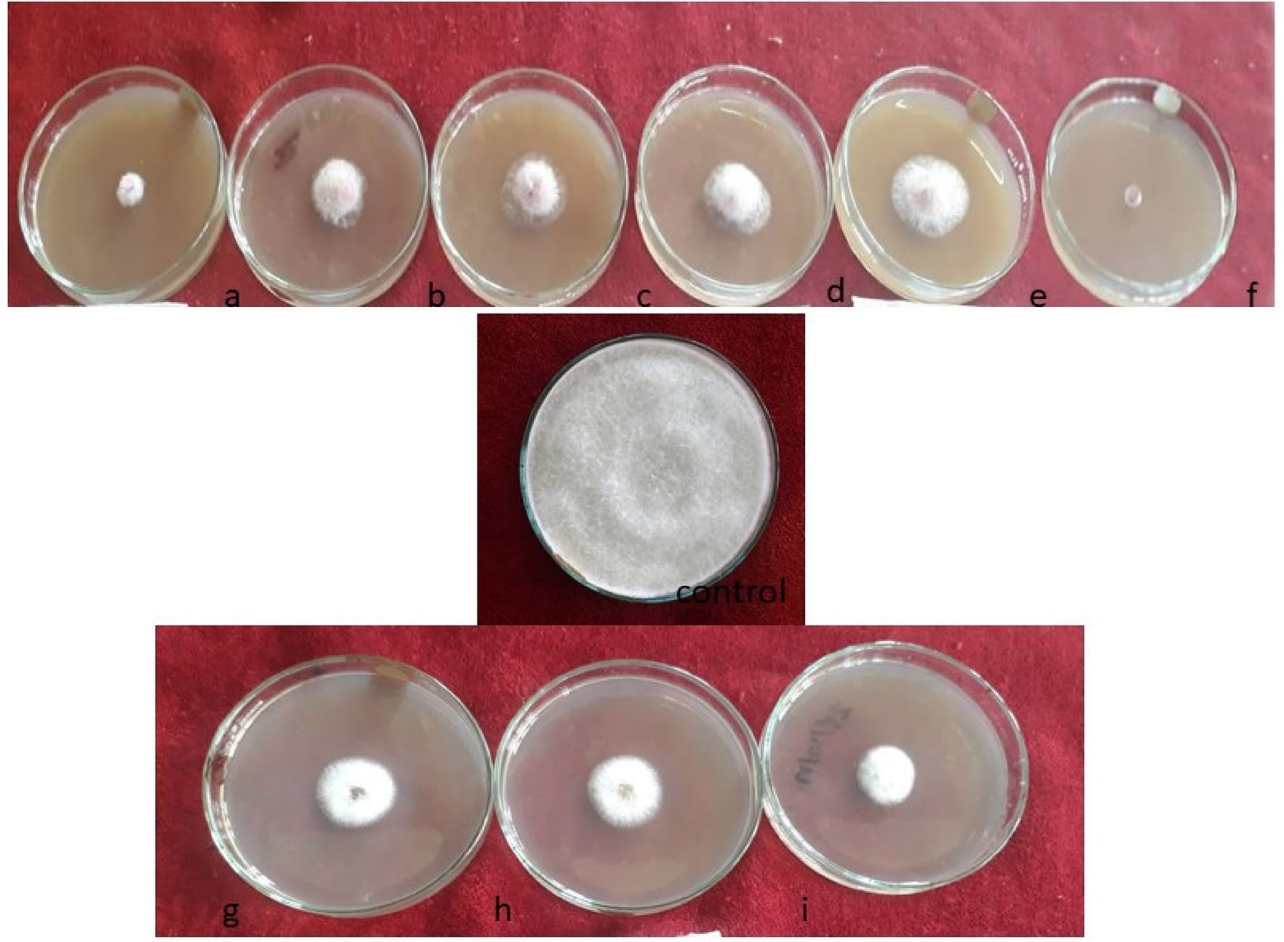
In vitro efficacy of fungicides against pathogen mycelial growth. (**a**) Captan50WP, (**b**) Tebuconazole 25EC, (**c**) Mancozeb 75WP, (**d**) Hexaconazole 75EC, (**e**) Metalaxyl 72WP, (**f**) Carbendazim 50WP, (**g**) Metiram + pyraclostrobin 60WG, (**h**) Difenconazole25EC, (**i**) Kresoximmethyl 44.3 SS.

All the nine fungicides when tested in vitro through poisoned food technique were significantly superior over control in inhibiting the mycelial growth of the fungus (Table 3, Fig. 5). However, carbendazim 50 WP at the rate of 250 ppm proved most effective with minimum colony diameter (7.33 mm) as compared to check. The pathogen recorded a colony diameter of 10.33, 22.00, 21.33, 22.33, 22.33, 23.33, 24.66 and 29.00 mm in Petri plates containing captan 50 WP at the rate of 1500 ppm, difenoconazole 25 EC at the rate of 250 ppm, metiram + pyraclostrobin 60 WG at the rate of 250 ppm, tebuconazole 75 WG at the rate of 250 ppm, kresoxim methyl 44.3 SS at the rate of 250 ppm, mancozeb 75 WP at the rate of 1500 ppm, hexaconazole 5 EC at the rate of 250 ppm and Metalaxyl MZ 72WP at the rate of 250 ppm, respectively. Similar inferences were drawn by Sharma and Ratnoo^26^, Somu et al.^27^ and Kumar and Mane^28^. The fungicides can have a varied effect on the growth of pathogens. Fungicides can interfere with the membrane system of microbes, thereby hindering their growth^29^. Further, certain fungicides can cause hydrolysis of phospholipids into free acids, leading to lysis in fungi^30^. Carbendazim is well known for its interference with the mycelial growth and affecting conidia formation and spore germination by stopping nuclear division^31^.

**Table 3:** In vitro efficacy of fungicides on mycelial growth inhibition of *Fusarium solani.*

S. No	Fungicide	Concentration (ppm)	Colony diameter (mm)	Mycelial growth of pathogen over check (%)
1	Tebuconazole 75WG	250	22.33	72.76
2	Difenoconazole 25EC	250	22.00	73.17
3	Hexaconazole 5EC	250	24.66	69.92
4	Metalaxyl MZ 72WP	250	29.00	64.63
5	Kresoxim methyl 44.3SS	250	22.33	72.76
6	Carbendazim 50WP	250	7.33	91.06
7	Metiram (55%) + pyraclostrobin(5%) 60WG	250	21.33	73.98
8	Captan 50WP	1500	10.33	87.40
9	Mancozeb 75WP	1500	23.33	71.54
10	Control	–	82.00	

C.D. (*p* ≤ 0.05) = 2.05.

### Field experiment

It is evident in Tables 4 and 5, seeds sown in raised beds and treated with carbendazim showed the lowest disease incidence (10.97%) and intensity (2.89%) followed by carbendazim treated ridge sowing with disease incidence and intensity of 16.29% and 4.89%, respectively. *Trichoderma harzianum* treated seeds and sown in raised beds showed disease incidence (22.14%) and intensity (6.90%) followed by *T. harzianum* treated seeds sown in ridges showing the disease incidence of 26.33 per cent and intensity (8.00%). Seeds sown in flat beds and treated either with carbendazim or *T. harzianum* showed poor results though significantly better from untreated seeds sown in flat beds. Untreated plots recorded the highest disease incidence (65.06%) and intensity (33.29%). The reason behind lower disease in raised beds and ridges was the controlled moisture levels. The results were in agreement with the results of Singh et al.^32^ who concluded that the root rot infected pea fields exhibited severe disease incidence of 60 per cent. Ketta et al.^33^ also concluded that the seed treatment with carbendazim exhibited minimum disease incidence followed by seed treatment with *T. harzianum*. Habtegebriel and Boydom^34^, reported that sowing on raised beds significantly reduced the incidence of root rot of faba bean than sowing on flat beds (cross reference).

**Table 4:** Effect of seed treatment and planting methods on disease incidence (%) of root rot of pea under field conditions.

Seed treatment	Disease incidence (%)			
	Planting methods			
	Raised beds	Ridges	Flat beds	Mean
Carbendazim @ 0.1%	10.97 (3.46) *	16.29 (4.15)	33.05 (5.83)	20.10 (5.09)
*Trichoderma harzianum* 10^9^ cfu	22.14 (4.81)	26.33 (5.22)	37.23 (6.18)	28.56 (6.82)
Sterile distilled water	48.37 (7.02)	56.84 (7.60)	65.06 (8.12)	56.75 (6.05)
Mean	27.16 (4.59)	33.15 (6.82)	45.11 (6.05)	

*Figures in parenthesis are square transformed values.

C.D (*p* ≤ 0.05).

Planting method (M): 0.103.

Seed treatment (S): 0.103.

(M × S): 0.179.

**Table 5:** Effect of seed treatment and planting methods on disease intensity (%) of root rot of pea under field conditions.

Seed treatment	Disease intensity (%)			
	Planting methods			
	Raised beds	Ridges	Flat beds	Mean
Carbendazim @ 0.1%	2.89* (1.97)	4.89 (2.26)	10.12 (3.41)	7.63 (2.80)
*Trichoderma harzianum* 10^9^ cfu	6.90 (2.79)	8.00 (2.92)	12.11 (3.62)	11.76 (3.34)
Sterile distilled water	13.11 (3.65)	22.39 (4.83)	33.29 (5.85)	18.50 (4.29)
Mean	5.96 (2.55)	9.00 (3.11)	22.93 (4.78)	

*Figures in parenthesis are square transformed values.

C.D (*p* ≤ 0.05).

Planting method (M): 0.184.

Seed treatment (S): 0.184.

(M × S): 0.319.

As evident in Table 6, the pod yield varied significantly with disease levels which in turn varied with planting methods and seed treatments. Carbendazim treated seeds in raised beds and ridges supported higher pod yield of 89.63 q ha^−1^ and 81.74 q ha^−1^, respectively, although, significantly higher than control and flat beds. *T. harzianum* treated seeds in raised beds and ridges recorded pod yield of 73.27 q ha^−1^ and 71.04 q ha^−1^, respectively. Flat beds which suffered higher levels of disease had poor yield irrespective of seed treatments. The results were in agreement with Pooniya et al.^35^ who attributed the high yield to the control of disease by using different treatments. Du et al.^36^ also reported higher yields and disease control in plants sown in raised beds, ridges and furrows than in flat beds.

**Table 6:** Effect of seed treatment and planting methods on fresh pod yield (q ha^−1^) of root rot of pea under field conditions.

Seed treatment	Fresh pod yield (q ha^−1^)			
	Planting methods			
	Raised beds	Ridges	Flat beds	Mean
Carbendazim @ 0.1%	89.63	81.74	69.08	80.06
*Trichoderma harzianum* 10^9^ cfu	73.27	71.04	67.94	70.73
Sterile distilled water	61.74	59.33	55.17	58.74
Mean	74.86	70.70	64.06	

C.D (*p* ≤ 0.05).

Planting method (M): 0.726.

Seed treatment (S): 0.726.

(M × S): 1.257.

## Conclusion

Fungicides alone as a disease control option have reduced the disease intensity and incidence to a very great extent, but the pathogens are developing resistance against these chemicals. Further, the fungicides can cause severe effects like carcinoma in humans if drift hazard happens while spraying. Bio magnification of these chemicals in environment and soil is another threat. So, researchers are exploring other avenues, by which diseases can be controlled without having harm on humans, beneficial micro-organisms in soil and also reducing resistance in pathogens. Biocontrol agents and improved planting technique can be an effective means of disease management, owing to their low cost of production and safer approach. A successful merger of cultural, prophylactic and protective measures can pave a way for integrated and successful management of diseases. Also in nearer future, the chemical fungicides can be completely eliminated and safer approaches can begin a new era of plant pathology. As also in our current study, *Trichoderma harzianum* along with cultural practices have reduced disease in pea plants significantly, hence, the disease management module can be prepared by integrating these two components together successfully.

## Data Availability

All data generated or analyzed during this study are included in this article.

## References

[1] Ali I, Patil RV, Dharmatti PR, Sridevi O (2018). Study on genetic parameters and performance of garden pea (*Pisum sativum* L.) genotypes for yield and its components (under northern transitional belt of Karnataka, India). Int. J. Curr. Microbiol. Appl. Sci..

[2] Chatterton S (2019). Importance and causal agents of root rot on field pea and lentil on the Canadian prairies, 2014–2017. Can. J. Plant Pathol..

[3] El-Saadony MT (2021). The use of biological selenium nanoparticles to suppress *Triticum aestivum* L. crown and root rot diseases induced by Fusarium species and improve yield under drought and heat stress. Saudi J. Biol. Sci..

[4] Sukapura RS, Bhide VP, Patel MK (1957). Fusarium wilt of garden peas in Bombay state. Indian Phtyopathol..

[5] Masoodi SD, Bhat NA, Shah TA (2000). Occurrence and severity of root rot of peas (*Pisum sativum* L.) in Kashmir valley. SKUAST J. Res..

[6] Ferguson JN, Tidy AC, Murchie EH, Wilson ZA (2021). The potential of resilient carbon dynamics for stabilizing crop reproductive development and productivity during heat stress. Plant Cell Environ..

[7] Šišić A (2018). The ‘forma specialis’ issue in Fusarium: A case study in *Fusarium solani* f. sp. pisi. Sci. Rep..

[8] Yang T, Lupwayi N, Marc SA, Siddique KH, Bainard LD (2021). Anthropogenic drivers of soil microbial communities and impacts on soil biological functions in agroecosystems. Glob. Ecol. Conserv..

[9] Mamine F, Farès MH (2020). Barriers and levers to developing wheat–pea intercropping in Europe: A review. Sustainability.

[10] Deng W, Misra GM, Baker CA, Gibson KE (2021). Persistence and transfer of foodborne pathogens to sunflower and pea shoot microgreens during production in soil-free cultivation matrix. Horticulturae.

[11] Nisa RT (2021). Identification and characterization of triple action bioagents (TAB) and their potency against Fusarium wilt of lentil. Horticulturae.

[12] Xing Y, Harper WF (2020). Bacillus spore awakening: Recent discoveries and technological developments. Curr. Opin. Biotechnol..

[13] Barnett HL, Hunter BB (1987). Illustrated Genera of Imperfect Fungi.

[14] Aksoy, E. *et al*. Recent advances in potato (*Solanum tuberosum* L.) breeding. In *Advances in Plant Breeding Strategies: Vegetable Crops* (eds Al-Khayri, J. M. Jain S. M. & Johnson D. V.) 409–487 (Springer, 2021).

[15] El-Dawy EGAEM, Gherbawy YA, Hussein MA (2021). Morphological, molecular characterization, plant pathogenicity and biocontrol of Cladosporium complex groups associated with faba beans. Sci. Rep..

[16] Win TT, Bo B, Malec P, Khan S, Fu P (2021). Newly isolated strain of *Trichoderma asperellum* from disease suppressive soil is a potential bio-control agent to suppress Fusarium soil borne fungal phytopathogens. J. Plant Pathol..

[17] Kipkoech C, Kinyuru JN, Imathiu S, Meyer-Rochow VB, Roos N (2021). In vitro study of cricket chitosan’s potential as a prebiotic and a promoter of probiotic microorganisms to control pathogenic bacteria in the human gut. Foods.

[18] Gunny AAN (2021). Microwave-assisted solvent-free extraction of essential oil from *Coleus aromaticus*: Anti-phytopathogenic potential for fruit post-harvesting. 3 Biotech.

[19] Porter LD, Pasche JS, Chen W, Harveson RM (2015). Isolation, identification, storage, pathogenicity tests, hosts, and geographic range of *Fusarium solani* f. Sp. pisi causing fusarium root rot of pea. Plant Health Prog..

[20] Kumari N, Thakur BR, Singh A (2016). Occurrence of pea root rot/wilt complex disease in Himachal Pradesh. Himachal J..

[21] Ahmad M, Raja V, Ahmad P, Rizvi G (2012). Bio management of root rot of pea (*Pisum sativum* L.) caused by *Fusarium solani* f. sp. *pisi*. Int. J. Curr. Res..

[22] Hamid A, Bhat NA, Sofi TA, Bhat KA, Asif M (2013). Management of root rot of pea (*Pisum sativum* L.) through bioagents. Afr. J. Microbiol. Res..

[23] Sood M (2020). Trichoderma: The “secrets” of a multitalented biocontrol agent. Plants.

[24] Abbey JA (2019). Biofungicides as alternative to synthetic fungicide control of grey mould (*Botrytis cinerea*)—Prospects and challenges. Biocontrol Sci. Tech..

[25] Zhu Z, Tian Z, Li J (2021). A Streptomyces morookaensis strain promotes plant growth and suppresses Fusarium wilt of banana. Trop. Plant Pathol..

[26] Sharma A, Ratnoo SR (2015). Integrated Management of Root Rot of Pea (*Pisum sativum* L.) caused by *Fusarium solani* (Mart) Apple and Wolleweber.

[27] Somu R, Thammaiah N, Swamy GS, Kulkarni MS, Devappa V (2014). *In vitro* evaluation of fungicides against *Fusarium oxysporum* f. sp. *cubense*. Int. J. Plant Prot..

[28] Kumar P, Mane SS (2017). Efficacy of fungicides and biocontrol agents against *Fusarium oxysporum* f. sp. *ciceri*. Int. J. Curr. Microbiol. Appl. Sci..

[29] Yang C, Hamel C, Vujanovic V, Gan Y (2011). Fungicide: Modes of action and possible impact on nontarget microorganisms. Int. Sch. Res. Not..

[30] Al-Askar AA (2021). Discovering *Penicillium polinicum* with high-lytic capacity on *Helianthus tuberosus* tubers: Oil-based preservation for mold management. Plants.

[31] Song XS (2021). The ASK1 gene regulates the sensitivity of *Fusarium graminearum* to carbendazim, conidiation and sexual production by combining with β2-tubulin. Curr. Genet..

[32] Singh A (2021). Optimized irrigation regime and planting technique improve yields and economics in aloe vera [*Aloe barbadensis* (Miller)]. Ind. Crops Prod..

[33] Ketta HA, Hewedy OAER (2021). Biological control of *Phaseolus vulgaris* and *Pisum sativum* root rot disease using Trichoderma species. Egypt. J. Biol. Pest Control.

[34] Habtegebriel, B., & Boydom, A. Integrated management of faba bean black root rot (*Fusarium solani*) through varietal resistance, drainage and adjustment of planting time.* J. Plant Pathol. Microbiol.*10.4172/2157-7471.1000363 (2016).

[35] Pooniya V (2021). Six years of conservation agriculture and nutrient management in maize–mustard rotation: Impact on soil properties, system productivity and profitability. Field Crop Res..

[36] Du X (2021). Raised bed planting reduces waterlogging and increases yield in wheat following rice. Field Crop Res..

